# Supplementary material for: Neural correlates of tactile hardness intensity perception during active grasping
Source: PeerJ. 2021 Aug 2;9:e11760. doi: 10.7717/peerj.11760 (PMC8340901; doi:10.7717/peerj.11760)
Supplement: Supplemental Information 1 — Results of two sample t-test of each stimuli pair. STD indicates standard deviation. H28-57: For the experiment, four different stimuli with different hardness intensities (28, 36, 45, and 57 a.u.) were prepared where a greater number indicated a harder stimulus (physical hardness intensity of each stimulus is denoted as H28, H36, H45, and H57).There was no significant difference of perceived difference of hardness in stimulus pairs by gender. [file peerj-09-11760-s001.docx]

| Pairs of Stimuli | H28-H28 | H36-H36 | H45-H45 | H57-H57 | H28-H36 | H36-H45 | H45-H57 | H28-H45 | H36-H57 | H28-H57 |
| --- | --- | --- | --- | --- | --- | --- | --- | --- | --- | --- |
| Male  (Mean, std) | (0.944, 1.286) | (1.125, 1.273) | (1.111, 1.184) | (0.969, 1.688) | (2.819, 1.284) | (2.964, 1.400) | (3.089, 1.504) | (5.279, 1.696) | (5.438, 1.724) | (8.250, 1.583) |
| Female  (Mean, std) | (0.625, 1.131) | (0.686, 1.718) | (1.281, 1.732) | (0.603, 1.756) | (2.568, 1.839) | (2.516, 1.919) | (2.522, 1.716) | (5.732, 2.245) | (5.772, 2.092) | (8.144, 1.737) |
| t-statistics  (t(82)) | 1.208 | 1.290 | -0.507 | 0.963 | 0.702 | 1.184 | 1.580 | -1.013 | -0.781 | 0.286 |
| p-value | 0.23 | 0.201 | 0.614 | 0.338 | 0.484 | 0.240 | 0.118 | 0.314 | 0.437 | 0.775 |

**Supplementary Table 1. Explorative analysis of potential gender effects.** Results of two sample t-test of each stimuli pair. STD indicates standard deviation. H28-57: For the experiment, four different stimuli with different hardness intensities (28, 36, 45, and 57 a.u.) were prepared where a greater number indicated a harder stimulus (physical hardness intensity of each stimulus is denoted as H28, H36, H45, and H57). There was no significant difference of perceived difference of hardness in stimulus pairs by gender.
