# Supplementary material for: Neural correlates of tactile hardness intensity perception during active grasping
Source: PeerJ. 2021 Aug 2;9:e11760. doi: 10.7717/peerj.11760 (PMC8340901; doi:10.7717/peerj.11760)
Supplement: Supplemental Information 2 — rpINS: posterior insula in the right hemisphere, rpCerebellum: right posterior lobe of the cerebellum, H28-57: For the experiment, four different stimuli with different hardness intensities (28, 36, 45, and 57 a.u.) were prepared where a greater number indicated a harder stimulus (physical hardness intensity of each stimulus is denoted as H28, H36, H45, and H57). [file peerj-09-11760-s002.docx]

| Stimuli | Percent Signal changes in rpINS | Percent Signal changes in rpCerebellum | Differences of Percent Signal changes  (rpIns-rpCerebellum) |
| --- | --- | --- | --- |
| H28 | -0.048 | 0.101 | -0.149 |
| H36 | -0.027 | 0.087 | -0.114 |
| H45 | -0.022 | 0.056 | -0.078 |
| H57 | -0.007 | 0.047 | -0.054 |

**Supplementary Table 2**. **Percent signal changes for each stimulus within the ROI.** rpINS: posterior insula in the right hemisphere, rpCerebellum: right posterior lobe of the cerebellum, H28-57: For the experiment, four different stimuli with different hardness intensities (28, 36, 45, and 57 a.u.) were prepared where a greater number indicated a harder stimulus (physical hardness intensity of each stimulus is denoted as H28, H36, H45, and H57).
