# Supplementary material for: Neural correlates of tactile hardness intensity perception during active grasping
Source: PeerJ. 2021 Aug 2;9:e11760. doi: 10.7717/peerj.11760 (PMC8340901; doi:10.7717/peerj.11760)
Supplement: Supplemental Information 4 [file peerj-09-11760-s004.docx]

|  | Right hemisphere | | | | |
| --- | --- | --- | --- | --- | --- |
|  | MNI coordinates | | |  | |
| Brain region | X | Y | Z | Z score | voxels |
| (a) Tracking the hardness positively | | | | | |
| Rolandic_Oper_R | 40 | -26 | 18 | 5.23 | 18 |
| (b) Tracking the hardness negatively | | | | | |
| Cerebelum_Crus2_R | 42 | -74 | -40 | 5.09 | 10 |
| Family-wise error (FWE) corrected (p<0.05) | | | | | |

**Supplementary Table 4. Activated brain regions from parametric modulation analysis.**
